# Preferred surgical strategies for bladder diverticular carcinoma

**DOI:** 10.1038/s41598-025-30477-5

**Published:** 2025-12-07

**Authors:** Yanle Ye, Qingxia Chen, Zhiyang Huang, Qi Huang

**Affiliations:** Department of Urology, The First Hospital of Quanzhou Affiliated to Fujian Medical University, No. 1028, Anji Road, Quanzhou, 362000 Fujian China

**Keywords:** Bladder cancer, Bladder diverticular carcinoma, Laparoscopic partial cystectomy, Cancer, Urology

## Abstract

To investigate the safety and efficacy of laparoscopic partial cystectomy in the treatment of bladder diverticular carcinoma. A retrospective analysis was performed on the clinical data of 12 patients diagnosed with bladder diverticular carcinoma who were treated at Quanzhou First Hospital Affiliated to Fujian Medical University between January 2016 and May 2023. All patients underwent laparoscopic partial cystectomy. The study assessed surgical parameters (e.g., operation time, intraoperative blood loss), pathological findings (tumor grade, stage, surgical margin status), postoperative complications, adjuvant treatment regimens, and long-term follow-up outcomes (tumor recurrence, survival status). All 12 operations were completed successfully without major intraoperative complications. Postoperative pathology confirmed bladder urothelial carcinoma in all cases, with negative surgical margins in all patients. Postoperatively, all patients received regular intravesical instillation chemotherapy. Additionally, 5 patients underwent systemic intravenous chemotherapy, and 1 patient received intra-arterial chemotherapy as adjuvant treatment to reduce the risk of tumor recurrence. The median follow-up duration was 16–49 months. During follow-up, 11 patients remained free of tumor recurrence, while 1 patient developed recurrent bladder cancer at 15 months postoperatively (subsequently managed with transurethral resection of bladder tumor and adjuvant radiotherapy, achieving long-term survival). Laparoscopic partial cystectomy is a safe and effective therapeutic option for bladder diverticular carcinoma, characterized by low postoperative complication rates and favorable tumor control. Larger-sample, multi-center studies with longer follow-up are needed to further validate its long-term prognostic benefits.

**Supplementary Information:**

The online version contains supplementary material available at 10.1038/s41598-025-30477-5.

## Introduction

Bladder diverticular carcinoma (BDC) is a rare malignant neoplasm arising from the bladder, accounting for approximately 0.8% to 10% of all bladder malignancies in clinical practice^1^. Owing to the structural characteristics of bladder diverticula—such as a relatively thin muscular layer, incomplete development, or even complete absence of the muscularis propria—tumors within the diverticulum exhibit a higher propensity for invasion or metastasis compared to conventional bladder tumors^2^. Traditional therapeutic modalities for BDC, including transurethral resection of bladder tumor (TURBT) and radical cystectomy^3^, have inherent limitations and drawbacks. For instance, TURBT often fails to achieve complete tumor resection due to the anatomical inaccessibility of diverticular lesions, while radical cystectomy is associated with high perioperative morbidity and a significant impact on patients’ quality of life. Thus, the safety and efficacy of these traditional approaches in BDC management remain to be further validated. To explore the safety and efficacy of laparoscopic partial cystectomy (LPC) for the treatment of BDC, we performed a retrospective analysis of 12 patients with BDC who underwent LPC at Quanzhou First Hospital Affiliated to Fujian Medical University between January 2016 and May 2023. This study focused on analyzing tumor features (e.g., grade, stage), diverticular morphology (e.g., size, location), surgical details (e.g., operative time, intraoperative blood loss), postoperative follow-up protocols, and clinical outcomes (e.g., complication rates, recurrence-free survival). The findings are presented as follows.

## Materials and methods

**Fig. 1:**
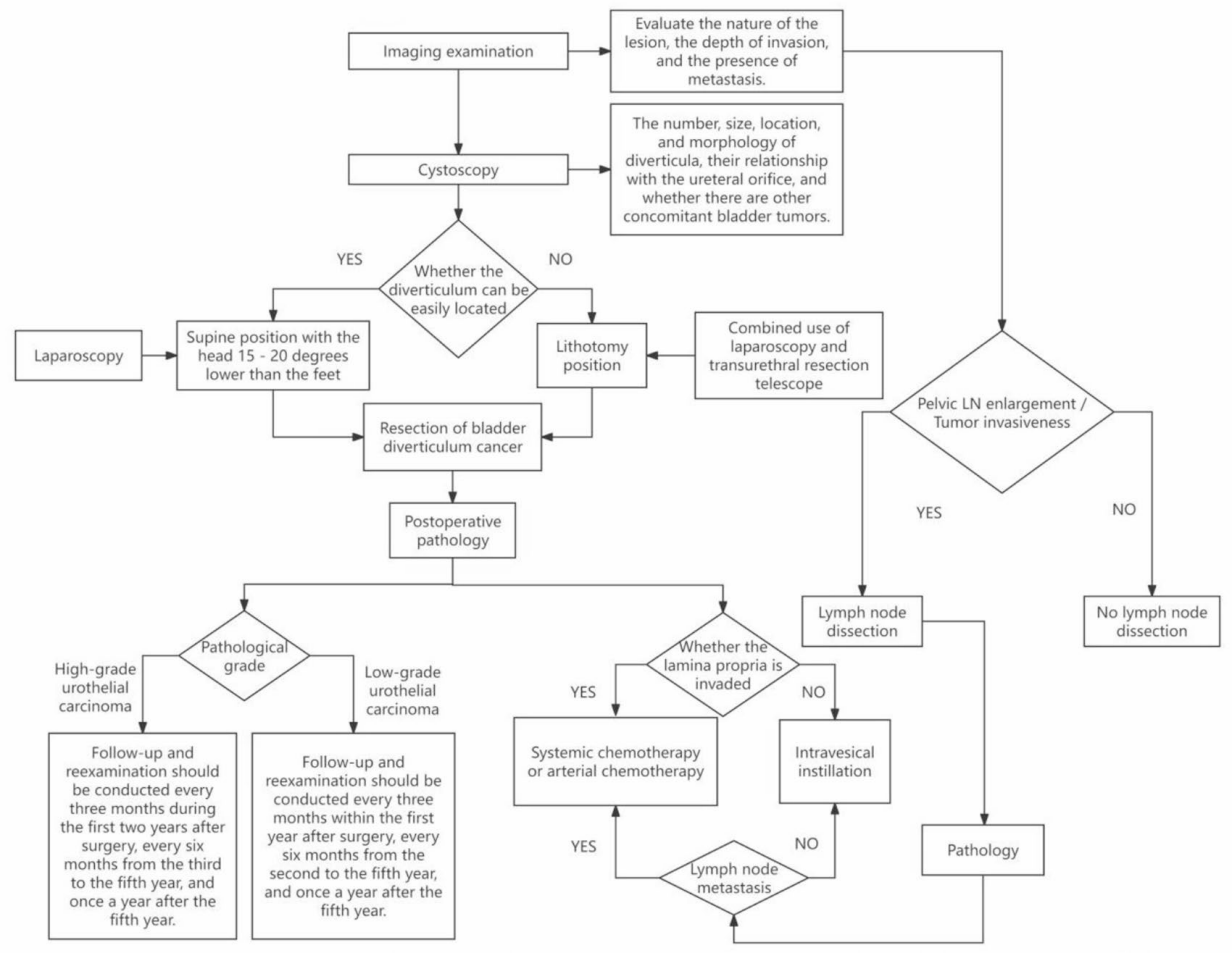
Treatment flowchart.

From January 2016 to May 2023, a total of 12 patients with BDC were admitted to Quanzhou First Hospital Affiliated to Fujian Medical University, all of whom underwent LPC.​ The treatment process is shown in Fig. 1.

All patients received general anesthesia. During the operation, most patients were placed in a supine position with the head down and feet elevated by 15–20° to optimize the surgical field of view and operating conditions, while those requiring combined resectoscope treatment were positioned in the lithotomy position. A three-port technique was adopted: the observation port was created below the umbilicus, and trocars were inserted at the sites 2 transverse fingers below the umbilicus and adjacent to the left and right rectus abdominis muscles as operating ports.​.

The surgical steps were performed as follows: First, the bladder was filled to locate the bladder diverticulum, followed by bladder emptying. A 0.8-cm incision was made at the upper edge of the diverticulum to access the bladder, and a suction device was inserted through this incision to completely aspirate residual urine. The diverticulum was resected along its margin, and the laparoscope was reinserted into the bladder to check for residual diverticular tumors. The bladder wall was sutured in two layers using 3 − 0 absorbable sutures. The surgical field was locally irrigated and soaked with water for injection combined with gemcitabine or epirubicin for 15 min. According to preoperative CT or MRI findings, if pelvic lymphadenopathy was present or the tumor was indicated to be invasive, concurrent pelvic lymph node resection and dissection were performed intraoperatively.

In cases where the bladder diverticulum was difficult to locate, the lithotomy position was used, and a combined dual-endoscopic approach (laparoscope plus transurethral resectoscope) was employed. The light source of the resectoscope was used to penetrate the bladder wall for accurate localization. Double-J stents were placed in two patients to facilitate intraoperative identification and protection of the ureteral orifices, minimizing the risk of ureteral injury. Ureteral reimplantation was not performed; if indicated (e.g., tumor involving the ureteral orifice), it can be conducted laparoscopically during tumor resection, with the magnified view enabling precise ureterovesical anastomosis.At the end of the operation, a 22Fr three-lumen urinary catheter was indwelled. If the urine appeared reddish, bladder irrigation was conducted until the urine color became clear.​.

All patients received regular intravesical chemotherapy with gemcitabine or epirubicin for 10–12 months postoperatively. Depending on the pathological depth of invasion, consideration was given to whether systemic chemotherapy or arterial chemotherapy should be administered.

The follow-up duration ranged from 16 to 79 months. Follow-up and reexamination items included routine blood tests, a complete biochemical panel, urinary system ultrasonography, computed tomography (CT), non-contrast chest CT, and transurethral cystoscopy. For patients with high-grade tumors: Follow-up and reexamination were conducted every 3 months in the first 2 years after surgery, every 6 months from year 3 to year 5, and annually after 5 years. For patients with low-grade tumors: Follow-up and reexamination were performed every 3 months within 1 year after surgery, every 6 months from year 2 to year 5, and annually after 5 years.

The study protocol was approved by the Ethics Committee of the First Affiliated Hospital of Quanzhou, Fujian Medical University (approval number: Quanyi Ethics [2021] No. 292). All methods were conducted in accordance with relevant guidelines and regulations, adhering to the principles outlined in the Declaration of Helsinki. Confidentiality and anonymity of the patients were ensured throughout the study.​.

## Results

### Postoperative outcomes and follow-up results

The baseline characteristics of the patients are presented in Table 1. All 12 surgeries were completed successfully. The operative time was (75 ± 25) minutes, and the intraoperative blood loss was (10 ± 6) ml. Postoperatively, patients resumed anal flatus and oral intake within 1–3 days, with a postoperative hospital stay of (4 ± 2) days. No complications such as abdominal pain or intestinal adhesion occurred.

**Table 1:** Clinical features of 12 patients with bladder diverticular Carcinoma.

Clinical characteristics	Specific data
Gender (M/F)	8 / 4
Age (years)	45–88 (mean 74)
Admission reasons	
Gross hematuria	10
Dysuria/interrupted micturition	3
Frequency/dysuria	2
I ncidental ultrasound	2
Comorbidities	
BPH with obstruction	5 (male)
Urethral stricture	2
Imaging	
Diverticular tumors	12
Diverticular calculi	1
Pelvic lymphadenopathy	2
Distant metastasis	0
Diverticulum (cystoscopy)	
Single	6
Left bladder wall	3
Right bladder wall	2
Dome–posterior wall	1
Multiple	6
Diameter	0.8–6 cm (mean 2.3 cm)
Tumor (cystoscopy)	
Single	9 (1 with satellite lesions)
Large diverticula with multiple tumors	2
Diameter	0.8–2.6 cm

Postoperative pathological results showed that all patients were diagnosed with bladder urothelial carcinoma, with no residual cancer cells at the surgical margins (negative margins). Among them: 7 cases were low-grade papillary urothelial carcinoma, 5 cases were high-grade papillary urothelial carcinoma (1 case complicated with squamous cell differentiation); tumor staging included 3 cases of pTa stage, 7 cases of pT1 stage, and 2 cases of pT3 stage. Immunohistochemical results revealed a Ki67 index of 39–85%, and 1 patient had pathologically positive obturator lymph nodes. More detailed pathological characteristics are provided in Supplementary Table 1.

The postoperative adjuvant treatment regimen was as follows: All patients received regular intravesical chemotherapy with gemcitabine or pirarubicin for 10–12 months. Additionally, 5 patients (4 with high-grade pathological types, 1 with low-grade pathology, and 1 with squamous differentiation) received systemic intravenous chemotherapy. The regimen used was the gemcitabine plus cisplatin combination (GC regimen) for a total of 6 cycles. One patient (with high-grade pathology) also received bilateral internal iliac artery chemotherapy (GC regimen) for 4 cycles.

Three patients with concomitant severe benign prostatic hyperplasia (BPH) underwent prostatectomy for treatment 2 months postoperatively. For patients with urethral stricture, regular urethral dilation was performed postoperatively to prevent and alleviate symptoms caused by urethral stricture, ensuring proper urinary tract patency. Patients with severe BPH underwent prostatic enucleation 2 months postoperatively; for patients with urethral stricture, regular postoperative urethral dilation was performed to prevent and relieve symptoms caused by urethral stricture and ensure unobstructed urinary tract after surgery.

During follow-up, 11 patients showed no tumor recurrence in regular cystoscopy, urinary system CT, and urinary ultrasonography. One patient experienced tumor recurrence at 15 months of follow-up. The initial pathological examination revealed high-grade urothelial carcinoma, with extension into the perivesical fat, negative surgical margins, and one positive lymph node in the left obturator region. Upon recurrence, TURBT was performed, and pathology confirmed high-grade urothelial carcinoma with muscular invasion. The patient declined radical cystectomy and subsequently received adjuvant radical radiotherapy (total dose: 60 Gy), and is currently alive with long-term survival.

### A representative case

**Fig. 2:**
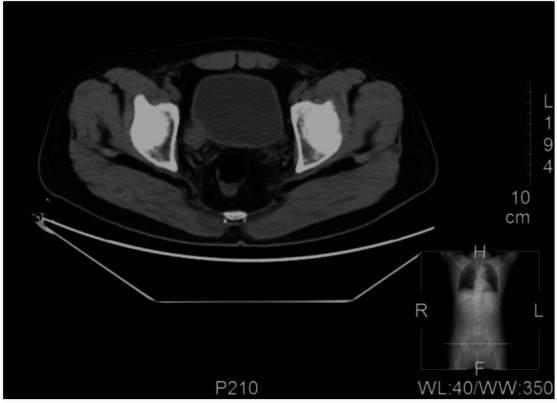
CT scan reveals a right lateral wall diverticulum of the bladder with a space-occupying lesion, measuring approximately 18 mm in diameter.

**Fig. 3:**
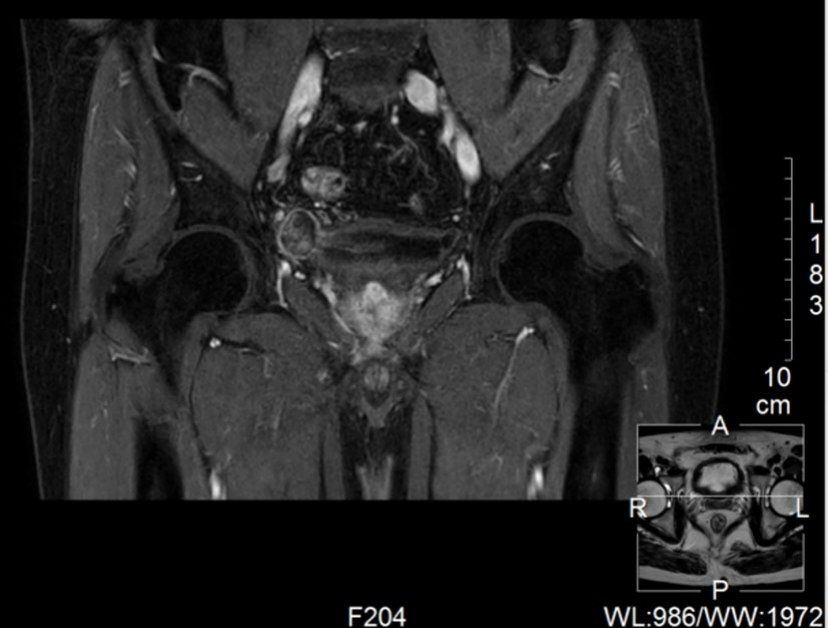
Magnetic Resonance Imaging (MRI) scan reveals a right lateral wall diverticulum of the bladder with a space-occupying lesion scan.

**Fig. 4:**
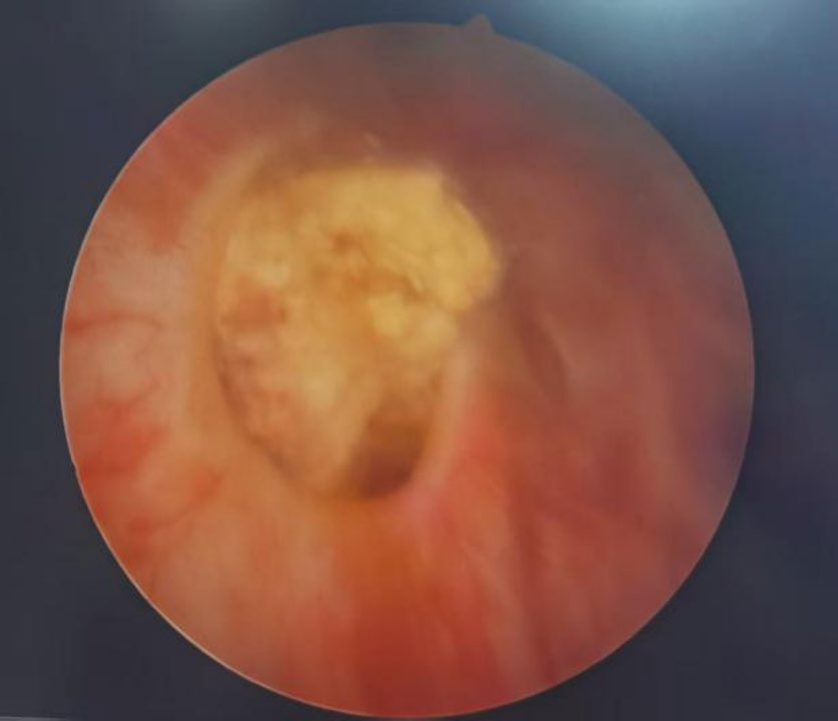
Preoperative cystoscopy revealed a diverticulum with a space-occupying lesion on the right lateral wall of the bladder, along with multiple small diverticula in the bladder.

**Fig. 5:**
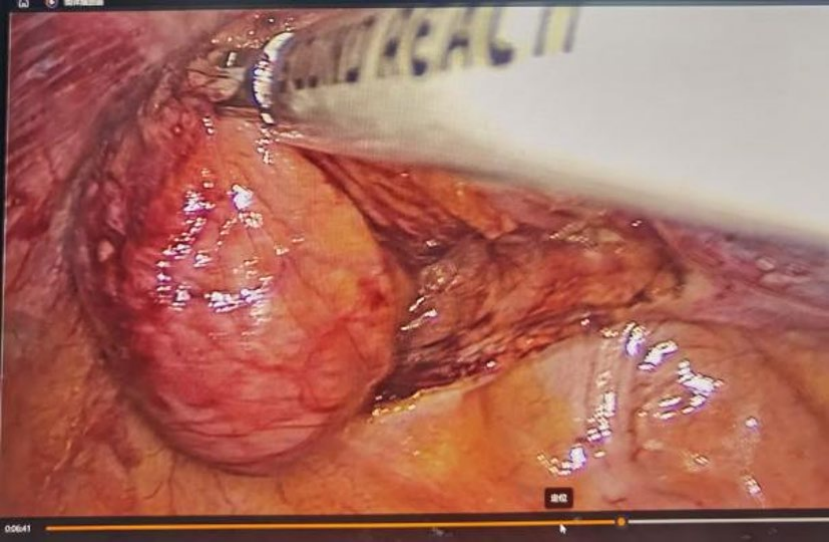
Intraoperative laparoscopic findings of the exophytic bladder diverticular lesion.

**Fig. 6:**
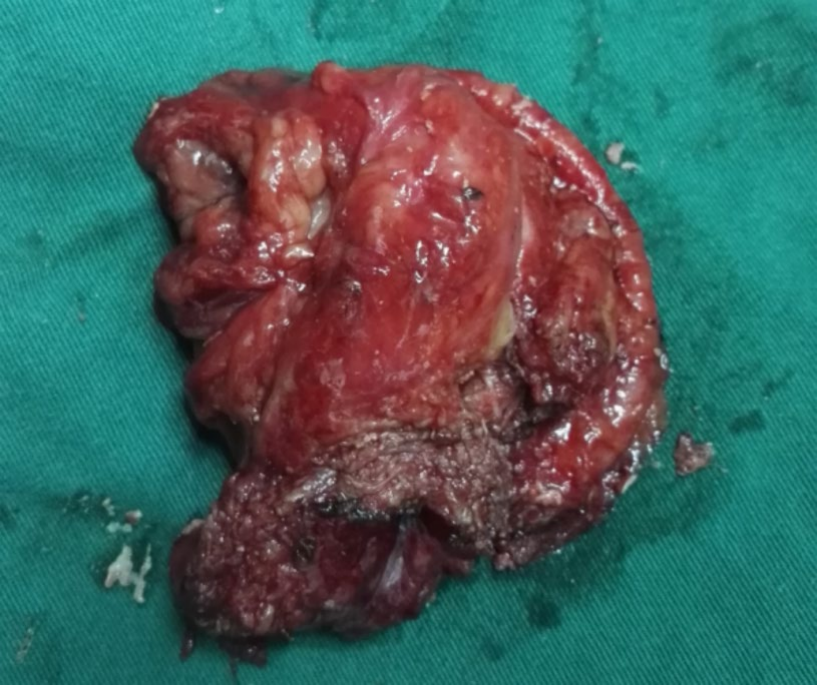
The resected lesion specimen.

**Fig. 7:**
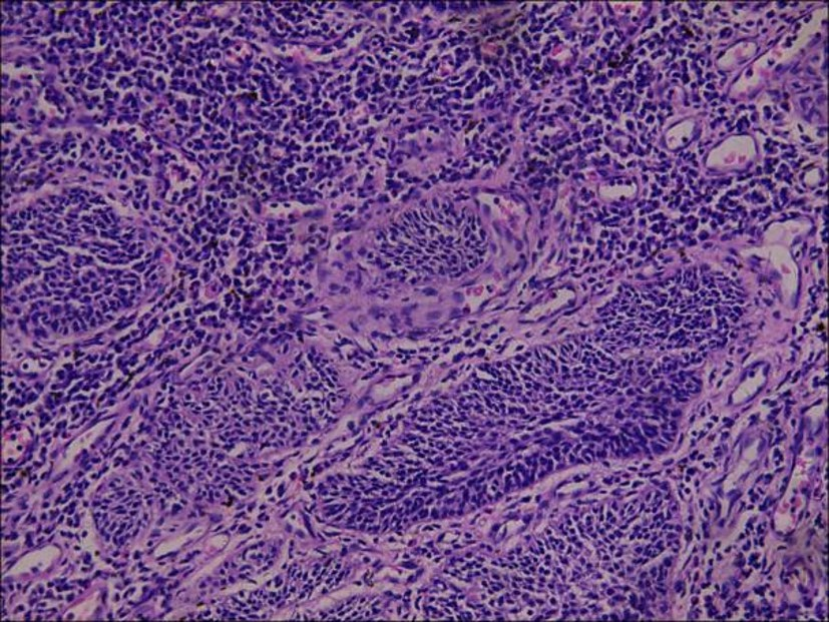
Postoperative Pathological Diagnosis: Bladder diverticulum with a mass inside. Combined with cytomorphological features and immunohistochemical findings, the lesion is consistent with high-grade urothelial carcinoma with squamous cell differentiation, accompanied by hemorrhage, necrosis, and focal calcification. The mass measures 1.5 × 1.5 × 1.3 cm and invades adipose tissue; no carcinoma is detected at the surgical margins. One right obturator lymph node: Negative (-) for carcinoma. Immunohistochemical results: P40 (+), CK5/6 (focal +), Gata-3 (focal +), CerbB2 (0), CK7 (focal weak +), CK20 (-), Ki67 (approximately 85% +).

**Fig. 8:**
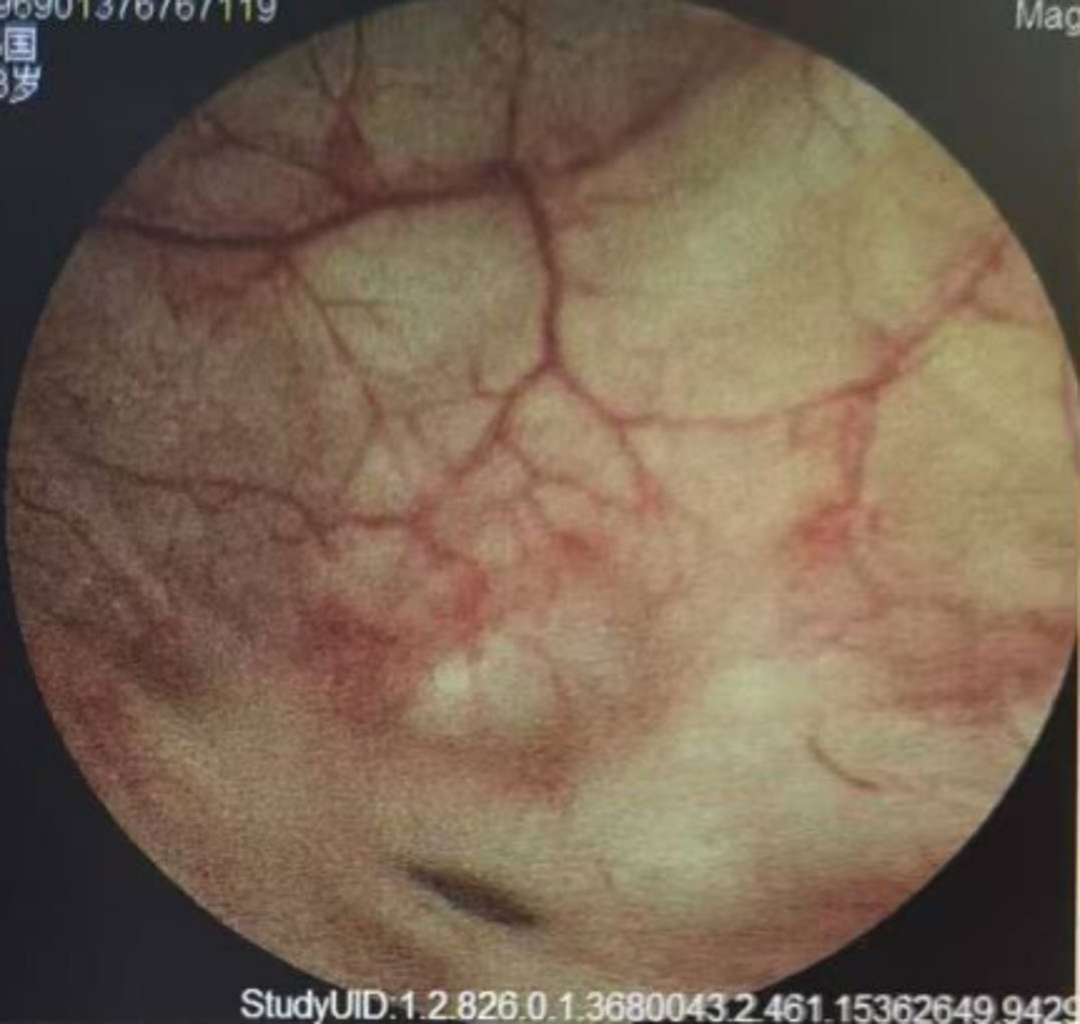
Cystoscopic findings at the 3-month postoperative follow-up: During the cystoscopic reexamination three months after the operation, the bladder showed good recovery with no evidence of tumor recurrence. Multiple small diverticula of the bladder were still present.

A 65-year-old male was identified with a bladder space-occupying lesion on abdominal ultrasonography during a routine physical examination one week prior to admission. The preoperative imaging findings, cystoscopic findings, intraoperative observations, postoperative pathological results, and follow-up cystoscopic findings are shown in Figs. 2, 3, 4, 5, 6, 7 and 8.

## Discussion

A bladder diverticulum is a pathological anatomical condition characterized by outpouchings of the bladder mucosa through the detrusor muscle fibers, forming irregularly shaped, variable-sized cavities that protrude from the bladder surface. It is classified into two types: congenital and acquired^4^.Congenital bladder diverticula are most common in children under 10 years of age, resulting from poor detrusor muscle development that leads to localized muscular layer weakness. The vast majority of congenital diverticula are located around the ureteral orifices^5^. Acquired bladder diverticula typically develop secondary to lower urinary tract obstruction. The underlying mechanism involves long-term exposure of the detrusor muscle to increased intravesical pressure, leading to decompensation; subsequently, localized weak areas of the muscular layer bulge outward under persistent high pressure. This type is more prevalent in elderly men, usually occurring on the lateral and posterior walls of the bladder and often accompanied by bladder trabeculation and cellules^6^.

Surrounded by the normal detrusor muscle of the bladder at its neck, the diverticulum presents a sac-like structure characterized by a “narrow neck and expanded base.” This anatomical feature, coupled with the low-pressure environment inside the diverticulum, impairs efficient urine emptying, thereby elevating the risks of lithiasis (stone formation) and urinary tract infection. Prolonged inflammatory stimulation may induce biochemical alterations in the mucosal lining of the diverticulum, which can further progress to bladder diverticular carcinoma^7,8^.

Previously, scholars reported that BDC is associated with a poor prognosis and short survival time^9^. However, recent studies have shown that BDC patients exhibit favorable postoperative follow-up outcomes and acceptable quality of life. Specifically, the 5-year cancer-specific survival rate is 83%±9% for non-invasive BDC (urothelial carcinoma) and 67%±7% for invasive BDC (urothelial carcinoma)^10,11^. This data highlights the clinical importance of early diagnosis and effective treatment for BDC to improve patient prognosis.

Bladder diverticulum carcinoma, like bladder cancer, originates from urothelial cells. The predominant pathological type in bladder diverticulum carcinoma is urothelial carcinoma (87.95%), followed by squamous cell carcinoma (5.22%). Small cell carcinoma, adenocarcinoma, and sarcoma are relatively rare^9,12,13^. The bladder diverticulum wall lacks a muscular layer and is composed only of the mucosal and submucosal layers, with a serosal layer separating the submucosal layer from the pelvic fat. Therefore, several scholars have suggested that bladder diverticulum carcinoma does not exhibit a T2 stage^10,14^, and any tumor extending beyond the serosal layer should be considered as T3 stage^15^. BDC typically exhibits a higher pathological grade compared to conventional bladder cancer. Owing to the anatomical features of bladder diverticula and chronic inflammatory stimulation, BDC often demonstrates more aggressive characteristics, with a relatively rapid disease progression^2^. It is worth noting that Golijanin et al.^10^ suggested that pT3 bladder diverticulum carcinoma, due to its incomplete invasive potential, may not necessarily have a worse prognosis compared to pT3 bladder cancer. However, due to the limited research data on bladder diverticulum carcinoma, further studies are needed to evaluate the relationship between pathological grading, pathological subtypes, and prognosis in bladder diverticulum carcinoma. In this study, all 12 cases of bladder diverticulum carcinoma were diagnosed as urothelial carcinoma, with 5 cases classified as high-grade papillary urothelial carcinoma and 1 case with squamous differentiation. Among the patients with squamous differentiation, the pathological diagnosis was pT3, with no signs of metastasis in the right obturator lymph nodes. Postoperatively, this patient received 6 cycles of systemic chemotherapy with the GC regimen, and no recurrence was observed during follow-up. Another pT3 patient had a positive lymph node biopsy in the left obturator region. After 15 months of follow-up, this patient experienced bladder tumor recurrence, for which TURBT was performed. Pathology revealed high-grade urothelial carcinoma with muscular invasion. After a second resection, the patient underwent adjuvant radical radiotherapy and is currently living with long-term survival.

Accurate staging of BDC may occasionally be compromised by insufficient specimen sampling. Therefore, obtaining adequate biopsy tissue during surgery or diagnostic procedures is essential to ensure diagnostic accuracy while maintaining patient safety. Laparoscopic partial cystectomy provides more complete specimens than transurethral resection of bladder tumors, enabling a more precise assessment of lesion characteristics and extent. This, in turn, facilitates more accurate diagnosis of malignant transformation and supports the development of scientifically sound and individualized treatment strategies. Additionally, we believe that even when urine cytology yields negative results, partial cystectomy should still be recommended because of the potential for false negatives. This approach allows for both definitive pathological confirmation and simultaneous management of the bladder diverticulum. In this study, no severe complications were observed in any of the 12 patients during the perioperative period.

TURBT, a classic bladder-sparing surgical modality for conventional bladder cancer^10^, is widely used in clinical practice. However, its application in BDC treatment is associated with several controversies and limitations, as outlined below: ① Due to the fragile anatomical structure of bladder diverticula, intraoperative bladder rupture or perforation is prone to occur, which may lead to tumor cell seeding and dissemination. Additionally, high-pressure bladder distension during surgery may cause extravesical tumor spread. ② The narrow neck of the diverticulum prevents the resectoscope from entering the diverticular cavity smoothly for precise manipulation, compromising the thoroughness and efficacy of the surgery. ③ Intraoperative electroresection may stimulate the obturator nerve, triggering involuntary muscle contractions. This can result in bladder injury or even perforation, increasing surgical risks.④ In the event of bladder perforation, intravesical instillation chemotherapy cannot be administered immediately postoperatively, which elevates the risk of intravesical tumor seeding and recurrence.⑤ Diverticula are often located in suboptimal positions for electroresection, making complete tumor resection difficult and potentially leaving residual lesions.⑥ During TURBT, the resulting tissue fragments are small, leading to insufficient pathological sampling. In addition, incomplete resection may further impair the accuracy of pathological diagnosis. ⑦ Patients with BDC have fewer opportunities for repeated TURBT and cannot receive subsequent treatments in the same manner as those undergoing TURBT for conventional bladder cancer. Thus, TURBT is not an ideal surgical approach for BDC.

Given the higher metastatic potential of bladder diverticular carcinoma (BDC), some scholars advocate radical cystectomy as the first-line treatment^2,9^. However, the authors argue that a more aggressive approach should be adopted for BDC staging and treatment, and recommend LPC as the preferred surgical modality. The specific rationale is as follows: ① Laparoscopic partial cystectomy enables complete resection of the diverticulum and tumor tissue, which facilitates accurate pathological staging and guides subsequent treatment. ② It avoids the need for urinary diversion surgery that is routinely required with radical cystectomy, thereby preserving patients’ quality of life. Additionally, compared with open partial cystectomy, LPC is less invasive, causes less pain, and promotes faster recovery, making it more acceptable to patients. ③ During surgery, initial bladder distension helps identify the location of the diverticulum; subsequent bladder emptying, followed by making a small incision at the upper edge of the diverticulum to access the bladder and aspirate residual urine, prevents urine extravasation outside the bladder. Combined with irrigation of the surgical field using water for injection mixed with epirubicin or gemcitabine, this reduces the risk of intra-abdominal tumor seeding and metastasis. ④ It allows simultaneous management of multiple diverticula or diverticular stones, improving surgical efficiency. ⑤ For cases with difficult diverticulum localization, the lithotomy position can be adopted, and a combined dual-endoscopic approach (laparoscope plus resectoscope) can be used. The light source of the resectoscope can penetrate the bladder wall to achieve accurate localization; if concurrent bladder satellite tumors are present, TURBT can be performed simultaneously. ⑥ Immediate intravesical instillation chemotherapy can be administered after partial cystectomy, further reducing the risk of intravesical tumor seeding and recurrence.

Given the high propensity of BDC for invasion and metastasis^9^, the authors advocate for postoperative adjuvant therapies based on pathological findings, including intravesical instillation chemotherapy, systemic intravenous chemotherapy, or intravesical arterial chemotherapy.

In this study, all patients exhibited negative surgical margins. Remarkably, all but one patient attained long - term recurrence - free survival. The sole patient who encountered recurrence underwent a second transurethral resection, followed by radical radiotherapy. Subsequently, this patient also achieved long - term survival. These results strongly substantiate the effectiveness of this treatment strategy in both preserving the bladder and attaining favorable therapeutic outcomes.

Elderly male patients with BDC are often complicated by BPH. The authors argue that tumor resection and transurethral resection of the prostate (TURP) should not be performed in the same surgical session. This is because concurrent surgery may increase surgical risks such as tumor seeding, bladder wall rupture or perforation, and bleeding-induced poor surgical field visualization. Furthermore, postoperative intravesical instillation of chemotherapeutic agents can irritate the surgical wound bed of the prostate, significantly exacerbating lower urinary tract symptoms (LUTS) such as urinary frequency, urgency, and dysuria. These severe symptoms may render patients unable to tolerate subsequent intravesical instillation therapy. Therefore, we recommend that surgery for BPH should be performed only after the completion of the first phase of intravesical instillation chemotherapy, which should be at least 2 months following bladder diverticular carcinoma surgery.

The present study indicates that LPC is safe and effective in the treatment of BDC. Compared with radical cystectomy or TURBT, LPC avoids the need for urinary diversion. Meanwhile, on the basis of complete resection of the tumor and diverticulum, it maximizes the patient’s quality of life. Additionally, this surgical approach reduces surgery-related risks and the incidence of perioperative complications, and shortens the patient’s hospital stay and recovery time. These advantages make laparoscopic partial cystectomy an ideal treatment option for bladder diverticular carcinoma.

## Supplementary Information

Below is the link to the electronic supplementary material.

Supplementary Material 1

## Data Availability

The datasets used and analysed during the current study can be made available from the corresponding author on reasonable request.
